# The new national clinical guideline for stroke: an opportunity to transform stroke care

**DOI:** 10.1016/j.clinme.2024.100025

**Published:** 2024-02-20

**Authors:** Ajay Bhalla, Louise Clark, Rebecca Fisher, Martin James

**Affiliations:** a Guy’s and St Thomas NHS Foundation Trust, Associate Clinical Director, King’s College London Stroke Programme, United Kingdom; b Stroke and Neurorehabilitation, Dorset County Hospital, Associate Clinical Director, King’s College London Stroke Programme, United Kingdom; c Clinical Policy Unit, NHS England, Associate Clinical Director, King’s College London Stroke Programme, United Kingdom; d Royal Devon University Healthcare NHS Foundation Trust, Honorary Clinical Professor, University of Exeter Medical School, Chair of the Intercollegiate Stroke Working Party, Clinical Director, King’s College London Stroke Programme, United Kingdom

## Introduction

In the UK, there are over 100,000 strokes occurring each year with approximately 1.3 million people living with stroke at an estimated cost of £26bn per year.^1^ With the changing demographics of an ageing population, there is a need to ensure that new developments in stroke care are delivered to reduce the growing burden of disability on individuals and society.

The new National Clinical Guideline for Stroke (2023) provides authoritative, evidence-based practice guidance to improve the quality of care delivered to every adult who has a stroke in the United Kingdom and Ireland.^2^ The guideline covers the management of stroke and transient ischaemic attack (TIA) in adults and is relevant to multidisciplinary staff, patients, families, carers and service planners. The guideline is relevant to general medical specialists who provide much of the acute stroke care, and given that 1 in 20 strokes occur in people already in hospital and clinicians in high-risk clinical areas (e.g., cardiology, renal ward and cardiothoracic units) should have a high level of awareness of acute stroke and time-critical nature of interventions.

The 2023 edition, like the previous editions of this guideline, has been overseen by the Intercollegiate Stroke Working Party, a group of senior representatives from the professional bodies, colleges and patient advocacy groups involved in stroke care. For this edition encompassing the whole of the UK and Ireland, the Working Party established a Guideline Development Group with representatives from the Working Party, from the Scottish Intercollegiate Guidelines Network (SIGN) and from the Irish National Clinical Programme for Stroke. The 2023 edition is a partial update of the 2016 edition and the scope was determined by generating 59 research questions from a multi-professional consultation. The guideline is accredited by NICE.

Depending on the strength of the evidence, recommendations in this guideline are either strong (a treatment or service ‘should be provided/offered’) or conditional (a treatment ‘should be considered’ or ‘may be considered’). This *Clinical Medicine Concise Guideline* summaries the key elements from the National Clinical Guideline for Stroke (2023) under the following subheadings.

1) Organisation of stroke services.
2) Management of TIA and minor stroke.
3) Intravenous thrombolysis.
4) Mechanical Thrombectomy.
5) Management of intracerebral haemorrhage.
6) Rehabilitation and Recovery.
7) Long term management and secondary prevention.

### Organisation of stroke services

Pre-hospital emergency services should be configured to maximise the delivery of timely acute treatments to the local population. Research examining the effects of mobile stroke units and direct transfer to thrombectomy centres in the context of UK healthcare is in progress. The mainstay of treatment for acute stroke at a population level, remains specialist stroke unit care resulting in saving lives, reducing long-term brain damage, disability and healthcare costs.^3^ The Guideline makes recommendations for in-patient staffing levels to support the delivery of multidisciplinary care. Community stroke rehabilitation services, including delivery of early supported discharge should be provided in a timely way following hospital discharge. It is important that there is a robust governance infrastructure in place to monitor the quality of stroke services delivered.

1) Hyperacute, acute and rehabilitation stroke services should provide specialist medical, nursing and rehabilitation staffing levels matching the recommendations in Table 1. Rehabilitation should be available 7 days a week, with initial assessment taking place by OT/PT/SLT within 24 h of admission.

**Table 1:** Recommended levels of staffing for hyperacute, acute and rehabilitation units.

	PT WTE/ Five beds	OT WTE/ Five beds	SLT WTE/ Five beds	Psy WTE/ Five beds	Dietn WTE/ Five beds	Nurse WTE/ One bed	Consultant Physician
**HASU**	1.02	0.95	0.48	0.28	0.21	2.9	24/7 availability: minimum six thrombolysis trained physicians on rota
**ASU and SRU**	1.18	1.13	0.56	0.28	0.21	1.35	ASU: daily ward round with 7 day cover SRU: twice weekly ward round *

ASU = acute stroke unit, HASU = hyperacute stroke unit, SRU = stroke rehabilitation unit.

PT = physiotherapy, OT = occupational therapy, SLT = speech and language therapy, Psy = psychology.

Dietn= dietician, WTE = whole time equivalent * can be provided by non-medical stroke consultant.
2) A stroke rehabilitation unit should have access to a consultant specialising in stroke rehabilitation (medical or non-medical, i.e., nurse or therapist, where professional regulation permits).
3) A service providing early supported discharge and community stroke rehabilitation should comprise a multidisciplinary core team structure.
4) The intensity and duration of intervention provided by the community stroke rehabilitation team should be established between the stroke specialist and the person with stroke and be based on clinical need tailored to goals.

### TIA and minor stroke

Any patient with a fully resolved acute onset neurological syndrome that might be due to cerebrovascular disease needs urgent specialist assessment (within 24 h) to determine whether the cause is vascular, given the substantial risk of subsequent stroke after a TIA (between 2–4% at 48 h post onset). Treatment for secondary prevention should be initiated as soon as the diagnosis is confirmed.

5) Healthcare professionals should not use assessment tools for TIA such as the ABCD2 score for risk stratification, informing urgency of referral or subsequent treatment.
6) For patients with suspected TIA, MRI should be the principal brain imaging modality for detecting the presence and/or distribution of brain ischaemia.
7) Dual antiplatelet therapy with either aspirin and clopidogrel for 21 days, or aspirin and ticagrelor for 30 days, followed by long-term monotherapy, should be considered in patients presenting within 24 h of TIA and minor stroke. Consideration should be given to clopidogrel resistance with CYP2C19 loss of function allele for patients with recurrent TIA or stroke whilst taking clopidogrel.

### Intravenous thrombolysis

The use of advanced imaging techniques such as CT perfusion and MR imaging have increased the eligibility of such patients to be treated up to 9 h after stroke onset as well as ‘wake up stroke’. Tenecteplase is considered comparable to alteplase in terms of efficacy and safety. To maximise the population benefit from thrombolysis, a renewed focus needs to be directed towards improving thrombolysis rates (currently between 10–11%) as well as door to needle times.^4^

8) Patients with ischaemic stroke in whom treatment can be started within 4.5 h of onset should be considered for thrombolysis with alteplase or tenecteplase.
9) Patients with acute ischaemic stroke who were last known to be well more than 4.5 h earlier should be considered for thrombolysis with alteplase if treatment can be commenced between 4.5–9 h of known onset, or within 9 h of the midpoint of sleep when they have woken with symptoms and have evidence of the potential to salvage brain tissue on CT perfusion or MRI (DWI-FLAIR mismatch).

### Mechanical thrombectomy

The principal advance in hyper-acute care has been thrombectomy delivered in an extended time window. This has been previously recommended up to 6 h after onset but now extends beyond 6 h and up to 24 h, and where the onset time is unknown. This is supported by a combination of approaches using a quantitative CT scan score (ASPECTS), CT angiography to identify large vessel occlusion as well as advanced imaging. Benefit also exists for thrombectomy for occlusions in the vertebrobasilar circulation. The challenge is for health services to support the delivery of thrombectomy to an increasing eligible population through extending 24/7 coverage in a networked approach.^5^ Estimates suggest that these recommendations will extend eligibility for thrombectomy to 15% of all strokes admitted in the UK.

10) Patients eligible for mechanical thrombectomy should receive prior intravenous thrombolysis as rapidly as possible (unless contraindicated), irrespective of whether they have presented to an acute stroke centre or a thrombectomy centre.
11) Patients presenting with acute anterior circulation ischaemic stroke and large artery occlusion between 6 and 24 h previously, including wake-up stroke, should receive mechanical thrombectomy on the basis of a combination of ASPECTS score and target or clinical imaging mismatch (CT/MR perfusion).
12) Patients presenting with acute ischaemic stroke in the posterior circulation within 12 h of onset should be considered for mechanical thrombectomy if they have a confirmed intracranial vertebral or basilar artery occlusion.

### Management of intracerebral haemorrhage (ICH)

Patients with ICH can deteriorate quickly and should be admitted directly to a hyperacute stroke unit for urgent specialist assessment and monitoring. Medical treatments for intracerebral haemorrhage have been challenging, resulting in poor therapeutic outcomes, but acute interventions directed at controlling physiological parameters such as blood pressure and anticoagulation reversal show promise, and surgical trials are ongoing. The focus of current recommendations is to limit haematoma expansion, prevent complications and establish the underlying cause.

13) Patients with acute spontaneous intracerebral haemorrhage with a systolic BP of 150–220 mmHg should be considered for urgent treatment within 6 h of symptom onset, aiming to achieve a systolic BP of 130–139 mmHg within one hour and sustained for at least 7 days.
14) Early non-invasive cerebral angiography (CTA/MRA within 48 h of onset) should be considered for patients with acute spontaneous intracerebral haemorrhage where a macrovascular cause is likely to be identified.

### Rehabilitation and recovery

There has been considerable expansion in rehabilitation research with detailed recommendations to inform patient centred, evidence-based practice. These cover motor recovery and the physical effects of stroke, the psychological complications as well as recommendations for those wishing to return to work. There has been a focus in on the importance of early assessment and treatment intensity with both language and motor recovery showing that greater amounts of therapy are associated with better recovery in line with updated NICE guidance (2023).^6^ Implementing innovative ways of providing rehabilitation through telerehabilitation and self-directed practice are also important.

15) People with motor recovery goals undergoing rehabilitation after a stroke should receive a minimum of 3 h of motor therapy a day at least 5 days out of 7 at an effective dose.
16) Healthcare professionals should select screening tools and assessments for psychological and cognitive problems appropriate to the needs of the person with stroke.
17) Intensive speech and language therapy (20–50 h of total therapy) may be considered from 3 months after stroke for those who can tolerate high-intensity therapy.
18) Healthcare professionals should use a validated measure in their assessment of post-stroke fatigue at regular intervals.
19) People with stroke should be considered to have the potential to benefit from rehabilitation at any point after their stroke based on need.

### Long term management and secondary prevention

Given that one quarter of all strokes are recurrent, it is vital that secondary prevention is delivered as soon as possible and continued long term through a comprehensive and personalised approach to multiple vascular risk factors. Lower targets for both lipids and blood pressure are supported by the evidence with a more intensive dose escalation required, usually in primary care, in line with updated NICE guidance on lipid management.^7^ Earlier initiation of anticoagulation has been recommended for patients with mild-moderate ischaemic stroke and atrial fibrillation but more trial evidence is required for a definitive answer in more severe stroke. There is also greater emphasis on physical activity to enhance cardiorespiratory fitness within a self-management framework.

20) Home blood pressure monitoring should be considered for guiding the management of BP-lowering treatment, with a typical home systolic BP target below 125 mmHg.
21) Lipid-lowering treatment for people with ischaemic stroke or TIA and evidence of atherosclerosis should aim to reduce fasting LDL-cholesterol below 1.8 mmol/L (equivalent to a non-fasting non-HDL-cholesterol below 2.5 mmol/L).
22) People with ischaemic stroke and atrial fibrillation or flutter should be considered for anticoagulation within 5 days of onset for mild stroke and may be considered for anticoagulation from 5–14 days of onset for moderate to severe stroke.
23) People who have an intracerebral haemorrhage whilst taking an antithrombotic medication to prevent vascular occlusive events may be considered for restarting antiplatelet treatment.
24) People below the age of 60 with ischaemic stroke or TIA of otherwise undetermined aetiology, in association with a patent foramen ovale (PFO) and a right-to-left shunt, should be considered for endovascular PFO device closure within 6 months of stroke.
25) People with stroke should be offered cardiorespiratory training or mixed training, for at least 30–40 min, three to five times a week for 10–20 weeks, guided by their goals and preferences.

### Limitations of the guideline

The 2023 edition is a partial update and not every recommendation has been reappraised, however out of 538 recommendations, almost 300 have been updated, added or endorsed since the 2016 edition.

### Implications for implementation

The implementation of this comprehensive update of stroke care across the entire pathway will present substantial challenges for workforce and other resources, but it offers the prospect of delivering substantial reductions in health and social care costs for the leading cause of adult-onset disability. Particular challenges arise with the full implementation of the rapidly developing evidence for mechanical thrombectomy, and in delivering rehabilitation at sufficient intensity and dose to reduce dependency and disability. Patient advocacy charities such as the Stroke Association are already providing strong voices demanding an acceleration of the uptake of thrombectomy for all those who need it, as well as improvements to life after stroke services. It is therefore important that organisations that plan, fund and deliver health and social care fully support the implementation of this guideline. Particular attention should be paid to the cost releasing impact of evidence-based stroke care, such as the impact on social care requirements significantly lessened by provision of intensive rehabilitation. Clinical networks play a crucial role in collaborating with providers and supporting service planners in quality improvement and service redesign particularly when working across geographical boundaries. National comparative audit through the Sentinel Stroke National Audit Programme (SSNAP) and its Scottish and Irish equivalents, measures the process of care and patient centred outcomes against quality standards derived from this guideline.^8^ Innovative approaches to deliver these recommendations have been highlighted ranging from improving selection for thrombectomy through artificial intelligence for advanced imaging, remote delivery of stroke rehabilitation as well as measuring the impact of longer-term support on patients and families. It is hoped that these recommendations underpinned by continuous quality improvement will offer the prospect of transforming stroke care in the coming years.

## Declaration of competing interest

There are no competing interests.
